# Analysis of Nucleotide Sequences of the 16S rRNA Gene of Novel *Escherichia coli* Strains Isolated from Feces of Human and Bali Cattle

**DOI:** 10.1155/2014/475754

**Published:** 2014-09-09

**Authors:** I Wayan Suardana

**Affiliations:** Department of Veterinary Public Health, Faculty of Veterinary Medicine, Udayana University, Jalan P.B. Sudirman, Denpasar, Bali 80232, Indonesia

## Abstract

Livestock especially cattle are known as a main reservoir of *Escherichia coli* O157:H7. This bacterium is considered as a pathogenic agent characterized by producing toxins, which are familiarly known as Shiga-like toxin-1 (Stx1) and Stx2. The aim of this work was to analyse the novel sequence of the 16S rRNA gene of strains isolated in this study in order to know the phylogenetic relationships between these sequences and those between the sequences of bacteria available in databanks. The results of this analysis showed that the strains KL-48(2) and SM25(1) that originated from human and cattle feces, respectively, are closely related among them and with respect to *E. coli* EDL 933, *E. coli* Sakai, *E. coli* ATCC 43894, *E. coli* O111:H-, *E. coli* O121:H19, *E. coli* O104:H4, and *Shigella sonnei* with more than 99% similarity values.

## 1. Introduction

The identification of pathogenic bacteria was traditionally performed by isolating the organism and studying it phenotypically by means of Gram staining and culture and biochemical methods, which has been the gold standard of bacterial identification [1].

With the invention of polymerase chain reaction (PCR) and automated DNA sequencing, the genome of some bacteria has been sequenced completely. A comparison of the genomic sequences of bacterial species showed that the 16S ribosomal RNA (rRNA) gene is highly conserved within a species and among species of the same genus and, hence, can be used as the new gold standard for the specification of bacteria [2]. To study bacterial phylogeny and taxonomy, the 16S rRNA gene sequences are very useful. With the gene presence in almost all bacteria, often existing as a multigene family, or operons, the function of the 16S rRNA gene over time has not changed, suggesting that random sequence changes are a more accurate measure of time and the 16S rRNA gene (1500 bp) is large enough for informatics purposes [3].

Using 16S rRNA sequences, numerous bacterial genera and species have been reclassified and renamed; classification of uncultivable bacteria has been made possible, phylogenetic relationships have been determined, and the discovery and classification of novel bacterial species have been facilitated [4]. This method has been successful in identifying Enterobacteriaceae species from a bone marrow transplant recipient [2], and the use of this method to identify or discover novel bacteria in clinical microbiology laboratories has successfully been reported also [4, 5].

*Escherichia coli* O157:H7 as one of enterohemorrhagic* Escherichia coli* (EHEC) are predominant strains causing infections to human. This disease ranges from simple diarrhea to the more complicated hemorrhagic colitis (HC) and hemolytic uremic syndrome (HUS) [6, 7]. Most infections caused by these bacteria are a result of the consumption of less cooked meat and unpasteurized dairy products and drinking water contaminated with feces [8]. In this study, we report the application of such technique to confirm novel* E. coli *strainsisolated from feces of human and Bali cattle and thus make the phylogenetic tree in order to know the relationship to each order sequence that is available in the databank. This study also intended to clarify previous study that identified that local isolates of* E. coli* O157:H7 that originated from animals and humans share genetic similarity coefficients [9, 10].

## 2. Materials and Methods

### 2.1. Bacterial Strains

Bacterial strains that were investigated in this study are, namely, SM-25(1) and KL-48(2). These strains were isolated from 80 feces samples of Bali cattle and 76 feces samples of humans suffering renal failure at the Sanglah General Hospital Centre, respectively. Both strains had been identified as serotype* E. coli* O157:H7 according to their genetic marker covering* stx1, stx2, *and* eae* gene [11, 12].

### 2.2. Extraction of DNA and PCR

DNA was extracted from bacterial strains using QIAamp DNA Mini Kits (Qiagen) according to manufacturer's instructions as described previously [11]. The 16S rRNA gene was amplified using Platinum PCR Supermix kit (Invitrogen) on Thermocycler Eppendorf Mastercycler personal/PTC 100. The PCR program was carried out in 40 *μ*L reaction volumes containing 2 *μ*L DNA template (300 ng/*μ*L), 34 *μ*L PCR Supermix 2x, and 2 *μ*L (20 pmol/*μ*L) of each primer. The primers were used in this study, that is, 27F (5′-AGAGTTTGATCCTGGCTCAG-3′) and U1492R (5′-GGTTACCTTGTTACGACTT-3′) [13]. The PCR amplification has initial DNA denaturation at 94°C for 5 min, followed by 35 cycles of denaturation at 94°C for 1 min, annealing at 55°C for 1 min, and elongation at 72°C for 1 min, which was followed by a final extension at 72°C for 5 min. 5 *μ*L PCR product was analyzed by electrophoresis (Bio-Rad) in 1% agarose (Gibco BRL) gel, at 90 volts for 45 min, followed by staining with 1% solution of ethidium bromide (50 *μ*L/L) and destaining with TBE 1x for 10 min. Gel was visualized by UV transillumination and recorded by digital camera FE-270 7.1 megapixels.

### 2.3. Sequencing and Phylogenetic Analysis

The sequencing of 16S rRNA gene was conducted using genetic analyzer (ABI Prism 3130 and 3130 xl Genetic Analyzer) at Eijkman Institute for Molecular Biology, Jakarta. The sequencing used both primers: Stx2 (F) and Stx2 (R). The sequences were edited to exclude the PCR primer binding sites and manually were corrected using MEGA 5.2 version software. The full gene sequences of strains KL-48(2) and SM-25(1) were compared automatically using the BLAST against the sequences of bacteria available in databanks (http://www.ncbi.nlm.nih.gov/). The phylogenetic analysis was constructed using neighbor-joining algorithm [14, 15].

### 2.4. Statistical Criteria for Species Identification

Identification of serotype was done through sequence similarity and/or difference nucleotides per total nucleotides. The criteria were determined based on the following: if the different nucleotides between the query and the study strain were 1–1.5% (14–22 bp), 1.5–5.0% (23–72 bp), and 5.0–7.0% (72–98 bp), the query strain should be given to the same species or genus or a different genus, respectively. Confirmation of strains was also determined based on the guidelines recommended by Janda and Abbott [16, 17].

### 2.5. GenBank Accession Number

The complete sequences (1380 bp) of the 16S rRNA gene of both strains KL-48(2) and SM-25(1) have been deposited in the International Nucleotide Sequence Database (INSD), that is, in the National Center for Biotechnology Information (NCBI).

## 3. Results and Discussion

**Figure 1:**
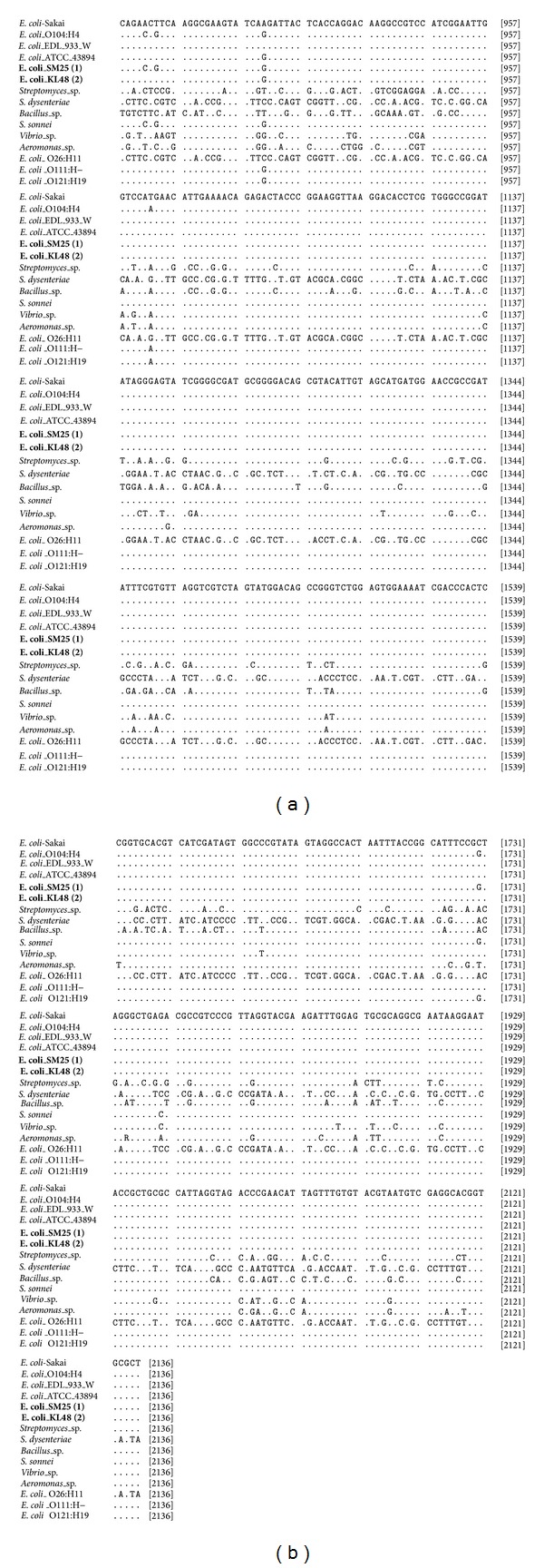
Nucleotides sequence of the 16S rRNA gene of the isolates* E. coli* SM-25(1) and* E. coli* KL-48(2) among nucleotides sequence of those available in databanks. Data indicated that the position of nucleotides is different among isolates and identical data for all isolates are not shown.

The analysis of 16S rRNA gene of* Escherichia coli* O157:H7 local isolates as an objective to be confirmed in this study has been successfully sequenced. Full sequences (1380 bp) of the 16S rRNA gene of both strains have been registered in GenBank with accession numbers KF768068 and KF768069 for strains SM-25(1) and KL-48(2), respectively. Alignment of the 16S rRNA gene of isolates* E. coli* SM-25(1) and* E. coli* KL-48(2) against some of those available in databanks is shown in Figure 1.

According to Figure 1, it showed some similarity or difference among nucleotides sequences that were aligned. Isolates* E. coli* SM-25(1) and* E. coli* KL-48(2) have tendency to show nucleotides sequence closely with isolates that originated from same species and distinctly for different species or genus. These results are propped by the ribosomal RNA sequencing as a more powerful technique for identification of bacteria, and these results agree with previous study. Patel [3] successfully uses 16S rRNA gene sequencing for bacterial pathogen identification in the clinical laboratory. Woo et al. [4] had used 16S rRNA gene sequencing for bacterial identification and discovery of novel bacteria in clinical microbiology laboratories, and Fattahi et al. [18] had developed the 16S rRNA as a PCR target for detection of* E. coli* in Rainbow Trout.

Furthermore, Patel [3] reported that the use of 16S rRNA gene sequence to study bacterial taxonomy has been used widely for a number of reasons. These reasons include (i) its presence in almost all bacteria, often existing as a multigene family or operons; (ii) the fact that the function of the 16S rRNA gene over time has not changed, suggesting that random sequence changes are a more accurate measure of time (evolution); and (iii) the fact that the 16S rRNA gene (1,500 bp) is large enough for informatics purposes.

The analysis of similarity or nucleotides different both* E. coli* SM-25(1) and KL-48(2) strains were studied against some strains of* E. coli,* that is,* E. coli* Sakai (BA000007),* E. coli* EDL 933 (AE005174),* E. coli* O104:H4 (AFOB02000112),* E. coli* O111:H-(GU237022),* E. coli* O121:H19 (JASV01000004),* E. coli* O26:H11 (AP010953), and* E. coli* ATCC 43894 as a bacterial control. The similarity analysis was also conducted on some strains of non-*E. coli,* that is,* Aeromonas* sp. (FM957460),* Vibrio* sp. (FM957459),* Shigella sonnei* (FR870445),* Streptomyces* sp. (AJ391832),* Bacillus* sp. (AB851799), and* Shigella dysenteriae* (CP000034) that are summarized in Table 1.

The data in Table 1 contain percentage of nucleotide similarity (lower-left triangle) and nucleotides difference/total nucleotides (upper-right triangle) of nucleotides analyzed. The summary of the 16S rRNA similarity analysis in Table 1 showed that* E. coli* KL-48(2) that originated from human feces has nucleotide similarity of 16S rRNA gene closely against some strains. These strains, that is,* E. coli* SM25(1),* E. coli* 121:H19,* E. coli* ATCC 43894,* E. coli* Sakai,* E. coli* EDL 933,* Shigella sonnei*,* E. coli* O111:H-, and* E. coli* O104:H4, are as high as 99.64, 99.56, 99.42, 99.35, 99.35, 99.35, 99.20, and 99.13%, respectively. Furthermore,* E. coli* SM-25(1) that originated from cattle feces also has high nucleotides similarity to the data of 16S rRNA that are available in GenBank also. It has nucleotides similarity to* Shigella sonnei*,* E. coli* O121:H19,* E. coli* O104:H4,* E. coli* ATCC 43894,* E. coli* Sakai, and* E. coli* EDL 933 as high as 99.71, 99.64, 99.49, 99.20, 99.13, and 99.13%, respectively. On the other hand, both strains showed percentage of nucleotide similarity distinctly to* Shigella dysentery*,* E. coli* O26:H11,* Bacillus* sp.,* Streptomyces* sp.,* Aeromonas*, and* Vibrio* sp.

Table 1 also showed that the number of nucleotides was different per total nucleotides among all isolates compared. Particularly for both isolates that were investigated, isolate KL-48(2) has few different nucleotides per total nucleotides against* E. coli* SM25(1),* E. coli* 121:H19,* E. coli* ATCC 43894,* E. coli* Sakai,* E. coli* EDL 933,* Shigella sonnei*,* E. coli* O111:H-, and* E. coli* O104:H4 as many as 5/1380, 6/1379, 8/1380, 9/1380, 9/1380, 9/1380, 11/1380, and 12/1379 nucleotides, respectively. Isolate SM-25(1) also has few different nucleotides per total nucleotides against* Shigella sonnei*,* E. coli* O121:H19,* E. coli* O104:H4,* E. coli* ATCC 43894,* E. coli* Sakai, and* E. coli* EDL 933 as many as 4/1380, 5/1379, 7/1379, 11/1380, 12/1380, and 12/1380 nucleotides, respectively.

Referred to the concept of similarity or nucleotides different between the query nucleotides and those compared, It is recommended when the sequences similarity is more than 90% or the nucleotides different between the query and those compared 1–1.5% (14–22 bp), the query should be categorized as the same species [16]. This assumption is supported by the similarity concept determined by Janda and Abbott [17]. The guideline recommends (i) the length of 16S rRNA gene should be sequenced minimum 500 to 525 bp and ideally 1,300 to 1,500 bp; (ii) criteria for species identification should be minimum >99% sequence similarity and ideally >99.5%. According to this guideline,* E. coli* SM-25(1) originated from feces of Bali cattle and* E. coli* KL-48(2) originated from human feces confirmed as the same species. This assumption was supported by the fact that both strains have nucleotides similarity of 99.64% or these strains have different nucleotides as many as 5/1380 nucleotides.

The high nucleotides similarity between 16S rRNA genes of isolates that originated from cattle and human made the conclusion the probability of the strain originated from feces of cattle as a main reservoir and then transmitted to human as a new host obvious occurred. The transmission of this bacterium from animals (cattle) to human can be facilitated by the consumption of meat that is less cooked or unpasteurized dairy products or drinking water contaminated with feces [19]. The results of the study all at once comes as a deep confirmation of previous study which identified that both isolates* E. coli* SM-25(1) and* E. coli* KL-48(2) share protein profile more than 70% [10], and the analysis using random amplified polymorphic DNA (RAPD) method indicated that both isolates also share genetic diversity more than 70% [9]. Moreover, analysis of phenogenotype of both isolates also had the same properties characterized. Both isolates genetically positive* eae* gene and the phenotypic study also showed either* E. coli* SM-25(1) or* E. coli* KL-48(2) had been colonize and causes cytophatic effects on vero cell. This study clarified both isolates had potency to colonize at the intestine host and induce attaching-effaching lesions [12].

The high nucleotides similarity (>99%) of both* E. coli* KL-48(2) and* E. coli* SM-25(1) strains with those of some nucleotides sequences that are available in GenBank also concludes that the tendency of both strains having virulent capacity as equal as of those, especially to the strains that are compared that is* E. coli* Sakai,* E. coli* EDL 933 and* E. coli* O104:H4, although there are needed to deep confirmations. on the other hand, the high similarity of* E. coli* SM-25(1) with* Shigella sonnei* that is 99.71% showed the probability of* E. coli* SM-25(1) as a novel strain outside of pathogenic* E. coli* strain especially to* Shigella sonnei* should be confirmed using the orther markers as a confirmation.

**Figure 2:**
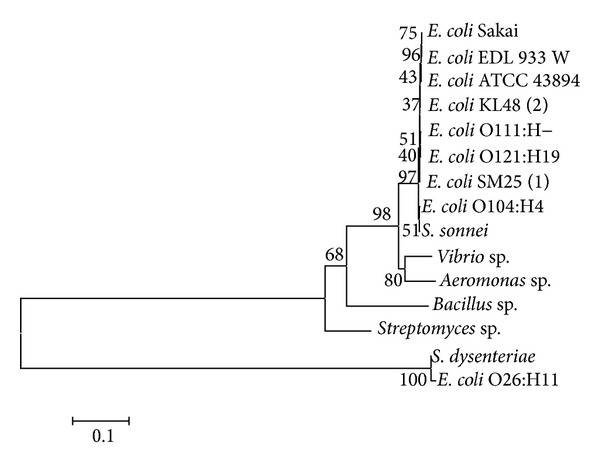
Phylogenetic tree was constructed using neighbor-joining algorithm [14] of nucleotides sequence of 16S rRNA gene. The number in the branch of phylogram indicates bootstrap value (%) by 1000-replication multiple, and scale indicates one per 1000 substitutions of nucleotides sequence of 16S rRNA gene.

Based on the data in Table 1, a phylogenetic tree of the 16S rRNA gene was performed using Clustal W programme in the MEGA 5.2 software. The phylogenetic tree was constructed using the neighbor-joining algorithm with bootstrap analysis for 1000 replicates (Figure 2).

Phylogenetic tree in Figure 2 showed that the* E. coli* KL-48(2) and SM25(1) performed close clade with some strains of pathogenic* E. coli* except for* E. coli* O26:H11. On the contrary, both strains also showed distinct clade against some strains that are available in databank. Some of those strains are* Streptomyces* sp. isolated from Yogyakarta,* Bacillus* sp. isolated from Jepara, and* Vibrio* sp.and* Aeromonas* sp. isolated from Lampung. As a result, both strains are proved to be a strain of pathogenic* E. coli* which potentially caused a serious outbreak of food borne illness equal to those strains that are characterized by bloody diarrhea and high frequency of serious complications including hemolytic-uremic syndrome (HUS).

## 4. Conclusion

The novel* Escherichia coli* strains SM-25(1) and KL-48(2) isolated from cattle feces and human feces, respectively, originated from the same source according to the analysis of 16S rRNA gene. These strains were predicted to have characteristics equal to* E. coli* Sakai,* E. coli* EDL 933,* E. coli* ATCC 43894,* E. coli* O111:H-,* E. coli* 121:H19,* E. coli* O104:H4, and* Shigella sonnei*.

## Figures and Tables

**Table 1:** Similarity analysis and nucleotides different among 16S rRNA genes using PHYDIT program.

	*Aeromonas *	*Vibrio* sp.	*E. coli *EDL 933 W	*E. coli* Sakai	*E. coli* ATCC 43894	*E. coli* O111:H—	*E. coli* O104:H4	*E. coli* O121:H19	*E. coli* SM25(1)∗	*E. coli* KL48(2)∗	*Shigella sonnei *	*Streptomyces* sp.	*Bacillus* sp.	*E. coli* O26:H11	*Shigella dysenteriae *
*Aeromonas *	—	158/1500	151/1376	151/1376	150/1376	154/1503	149/1375	153/1529	147/1376	148/1376	161/1529	329/1445	357/1377	817/1451	817/1451
*Vibrio* sp.	89.47	—	131/1380	131/1380	130/1380	134/1501	141/1379	146/1506	140/1380	138/1380	252/1642	326/1418	458/1528	810/1429	809/1429
*E. coli *EDL 933 W	89.03	90.51	—	0/1380	1/1380	6/1380	19/1379	13/1379	12/1380	9/1380	16/1380	282/1346	305/1325	739/1340	741/1340
*E. coli* Sakai	89.03	90.51	100.00	—	1/1380	6/1380	19/1379	13/1379	12/1380	9/1380	16/1380	282/1346	305/1325	739/1340	741/1340
*E. coli* ATCC 43894	89.10	90.58	99.93	99.93	—	5/1380	18/1379	12/1379	11/1380	8/1380	15/1380	282/1346	305/1325	739/1340	741/1340
*E. coli* O111:H—	89.75	91.07	99.57	99.57	99.64	—	15/1379	11/1506	14/1380	11/1380	24/1506	301/1420	338/1378	797/1424	797/1424
*E. coli* O104:H4	89.16	89.78	98.62	98.62	98.69	98.91	—	6/1378	7/1379	12/1379	5/1379	280/1345	305/1324	738/1339	740/1339
*E. coli* O121:H19	89.99	90.31	99.06	99.06	99.13	99.27	99.56	—	5/1379	6/1379	21/1539	309/1447	342/1383	808/1451	808/1451
*E. coli* SM25(1)∗	89.32	89.86	99.13	99.13	99.20	98.99	99.49	99.64	—	5/1380	4/1380	284/1346	304/1325	737/1340	739/1340
*E. coli* KL48(2)∗	89.24	90.00	99.35	99.35	99.42	99.20	99.13	99.56	99.64	—	9/1380	285/1346	307/1325	738/1340	740/1340
*Shigella sonnei *	89.47	84.65	98.84	98.84	98.91	98.41	99.64	98.64	99.71	99.35	—	314/1449	511/1601	813/1457	813/1457
*Streptomyces *sp.	77.23	77.01	79.05	79.05	79.05	78.80	79.18	78.65	78.90	78.83	78.33	—	278/1298	827/1427	829/1427
*Bacillus* sp.	74.07	70.03	76.98	76.98	76.98	75.47	76.96	75.27	77.06	76.83	68.08	78.58	—	768/1312	770/1312
*E. coli* O26:H11	43.69	43.32	44.85	44.85	44.85	44.03	44.88	44.31	45.00	44.93	44.20	42.05	41.46	—	17/1542
*Shigella dysenteriae *	43.69	43.39	44.70	44.70	44.70	44.03	44.73	44.31	44.85	44.78	44.20	41.91	41.31	98.90	—

*Strain in this study.
